# Palatal bone thickness at the implantation area of maxillary skeletal expander in adult patients with skeletal Class III malocclusion: a cone-beam computed tomography study

**DOI:** 10.1186/s12903-021-01489-0

**Published:** 2021-03-22

**Authors:** Weiting Chen, Kaili Zhang, Dongxu Liu

**Affiliations:** 1 Department of Orthodontics, School and Hospital of Stomatology, Cheeloo College of Medicine, Shandong University, No. 44-1 Wenhua Road West, Jinan, 250012 Shandong China; 2 Shandong Key Laboratory of Oral Tissue Regeneration, Jinan, China; 3 Shandong Engineering Laboratory for Dental Materials and Oral Tissue Regeneration, Jinan, China

**Keywords:** Class III malocclusion, Palate, Bone thickness, Maxillary skeletal expander, Cone-beam computed tomography

## Abstract

**Background:**

Maxillary skeletal expanders (MSE) is effective for the treatment of maxillary transverse deformity. The purpose of the study was to analyse the palatal bone thickness in the of MSE implantation in patients with skeletal class III malocclusion.

**Methods:**

A total of 80 adult patients (40 males, 40 females) with an average angle before treatment were divided into two groups, the skeletal class III malocclusion group and the skeletal I malocclusion group, based on sagittal facial type. Each group consisted of 40 patients, with a male to female ratio of 1:1. A cone-beam computed tomography scanner was employed to obtain DICOM data for all patients. The palatal bone thickness was measured at 45 sites with MIMICS 21.0 software, and SPSS 22.0 software was employed for statistical analysis. The bone thickness at different regions of the palate in the same group was analysed with one-way repeated measures ANOVA. Fisher’s least significant difference-t method was used for the comparison of pairs, and independent sample *t* test was employed to determine the significance of differences in the bone thickness at the same sites between the two groups.

**Results:**

Palatal bone thickness was greater in the middle region of the midline area (*P* < 0.01), while the thickness in the middle and lateral areas in both groups was generally lower (*P* < 0.001). The bone in the anterior, middle, and posterior regions of the two groups became increasingly thin from the middle area toward the parapalatine region. The palatal bone was significantly thinner in the area 9.0 mm before the transverse palatine suture in the midline area, 9.0 mm before and after the transverse palatine suture in the middle area, and 9.0 mm after the transverse palatine suture in the lateral area.

**Conclusion:**

The palatal bone was thinner in patients with class III malocclusion than in patients with class I malocclusion, with significant differences in some areas. The differences in bone thickness should be considered when MSE miniscrews are implanted. The anterior and middle palatal areas are safer for the implantation of miniscrews, while the thinness of the posterior palatal bone increases the risk of the miniscrews falling off and perforating.

## Background

Skeletal class III malocclusion, a common deformity, is caused by maxillary hypoplasia and/or mandibular hypergenesis [1]. The global prevalence of class III malocclusion in permanent dentition is 5.93% and varies greatly among and within different ethnic groups [2]. Tang assessed 108 Chinese male first-year dental students and found that the prevalence of Class III malocclusion was as high as 14.8% [3]. Patients with skeletal class III malocclusion often show transverse and sagittal abnormalities, and common clinical characteristics include maxillary transverse deformity (MTD), narrow width of the maxillary alveolar bone and/or maxillary dental arch, widened buccal corridor space when smiling, a v-shaped maxillary dental arch, and a unilateral or bilateral posterior crossbite, which affect oral function and maxillofacial attractiveness [4–6].

Maxillary arch expansion is an effective treatment for MTD [7, 8]. The emergence of bone-borne palatal expanders has enabled adults to expand their arches without surgery [9–11]. The simple structure of the palate, the tough palatal mucosa, and the low risk of root or blood vessel injury render the palatal bone a suitable area for the implantation of temporary skeletal anchorage devices (TSADs) [12, 13]. After a screw is implanted, the surface is mechanically embedded into the surrounding bone tissue such that a certain implant depth can effectively result in a larger contact area [14]. It has been reported that the bone thickness in the implant area is the key to maintaining the initial stability of miniscrew implants [15, 16]. Therefore, it is crucial to evaluate palatal bone thickness at the implant anchorage site.

Research has indicated the accuracy and reliability of cone-beam computed tomography (CBCT) for obtaining linear measurements, and the results obtained by using CBCT to measure palatal bone structure have been validated [17, 18]. Several studies based on CBCT have indicated sufficient bone density and good bone quality in the anterior region of the palatal bone, which has therefore been considered suitable for miniscrew implantation [19–22]. Moreover, researchers found that the bone thickness of the palate is related to age, sex, skeletal type, and other factors [18, 22–25]. However, the measurement range of palatal bone often do not include the complete implantation area of maxillary skeletal expanders (MSEs), and the specific type of adult skeletal class III malocclusion have not been studied. Therefore, in this study, the palatal bone thickness of patients with skeletal class III malocclusion, especially in the implantation area of the MSE, was analysed quantitatively, and the differences in palatal bone thickness between class III and class I malocclusion types were compared. Accordingly, the suitability of implantation sites for microimplants was explored, which can offer theoretical guidance for clinical anchorage implantation in patients with skeletal class III malocclusion.

## Methods

### Patients

**Fig. 1:**
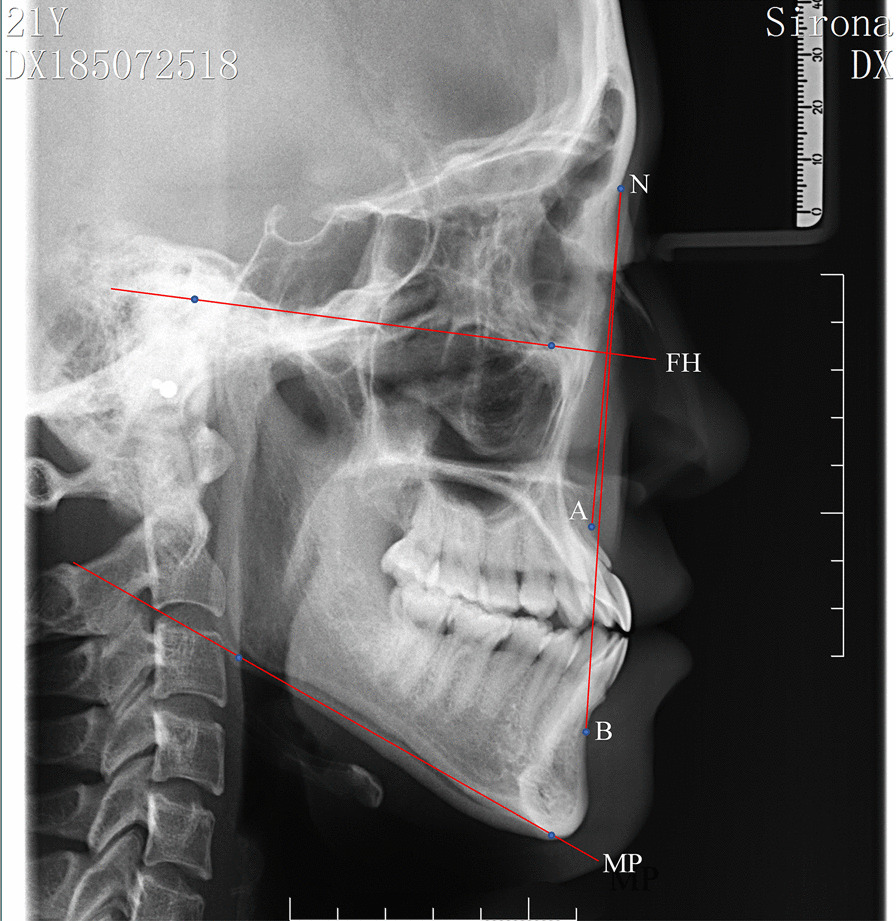
Schematic diagram of cephalometric measurement. MPA: The angle formed by FH (Frankfort horizontal plane) with MP (mandibular plane); ANB: The angle formed by point N (nasion), point A (subspinale), and point B (supramental)

All procedures performed in the present study involving human participants were approved by the Research Ethics Committee of Shandong University Dental School (Protocol No. 20201204) and were in accordance with the Declaration of Helsinki for research involving human subjects. The study was explained to the patients, and written informed consent for participation was obtained from them. Patients admitted to the orthodontic department of Shandong University Dental School from 2017 to 2020 were selected, and the CBCT data of the maxillofacial region in each participant were collected. The study inclusion criteria were as follows:

1. Patients with a spinal skeletal age of stage CS5 or CS6.
2. Patients with permanent dentition and no dentition defects (excluding third molars).
3. Patients with average angle. The mandibular plane angle (MPA) between the Frankfort horizontal plane and the mandibular plane was measured on a lateral cephalogram according to Downs analysis (Fig. 1), and the selection criterion of 22° ≤ MPA ≤ 32° was based on Chinese standards [26].
4. After CBCT scanning and clinical examination, no serious craniofacial, cleft lip or palate deformities were found, and there were no impacted teeth, supernumerary teeth, or jaw cysts in the measurement area.
5. Patients without a history of orthodontic treatment.
6. Patients without systemic diseases or other factors affecting bone metabolism.

All patients were were given detailed information about the study.

The lateral cephalogram confirmed the sagittal bone face type (Fig. 1). According to Steiner analysis [27], the patients were divided into the skeletal class III malocclusion group (ANB < 0.7°) and skeletal I malocclusion group (0.7° ≤ ANB ≤ 4.7°) and were numbered in the order of their first visit in each group. Patients were selected randomly using a random number table. The power calculation was performed to recruit the smallest needed sample size. The calculation was based on an α of 0.05 and a β of 0.2 to achieve a power of 80% and to detect the difference of 1 mm in palatal bone linear thickness measurements between groups, with a 1.47 mm estimated standard deviation [28]. The power analysis indicated a sample size of 35 in each group. To account for potential nonresponse, 40 patients were included in each group. The male to female ratio was 1:1 to exclude the influence of gender factors in each group. The mean and standard deviation of the age values in the class III malocclusion group and class I malocclusion group were 20.55 ± 3.81 years and 22.42 ± 4.58 years, respectively.

### CBCT scanning condition

All patients received CBCT before orthodontic treatment (NewTom 5G, QR srl, Verona, Italy; the layer thickness was 0.3 mm; the parameters were 110 kV and 5 mA). During scanning, the patients maintained the maximum occlusal contact, and their lips and tongues were relaxed without swallowing. The patients’ CBCT data were output in Digital Imaging and Communications in Medicine (DICOM) format and imported into Materialise Interactive Medical Image Control System (MIMICS, Version 21.0; Leuven, Belgium) software. A mask was then established, and the three-dimensional model was reconstructed and measured.

### Analytical method and content

The palate includes the hard palate and the soft palate. In this study, the hard palate (composed of the palatine process of the maxilla and horizontal plate of the palatine bone) was examined. In previous studies, the thickness of the palatal bone was analysed from front to back with the incisive foramina as the centre, which did not cover the whole posterior palatal area [22–25]. Therefore, in this study, the sutura palatina transversa was used as the centre.

**Fig. 2:**
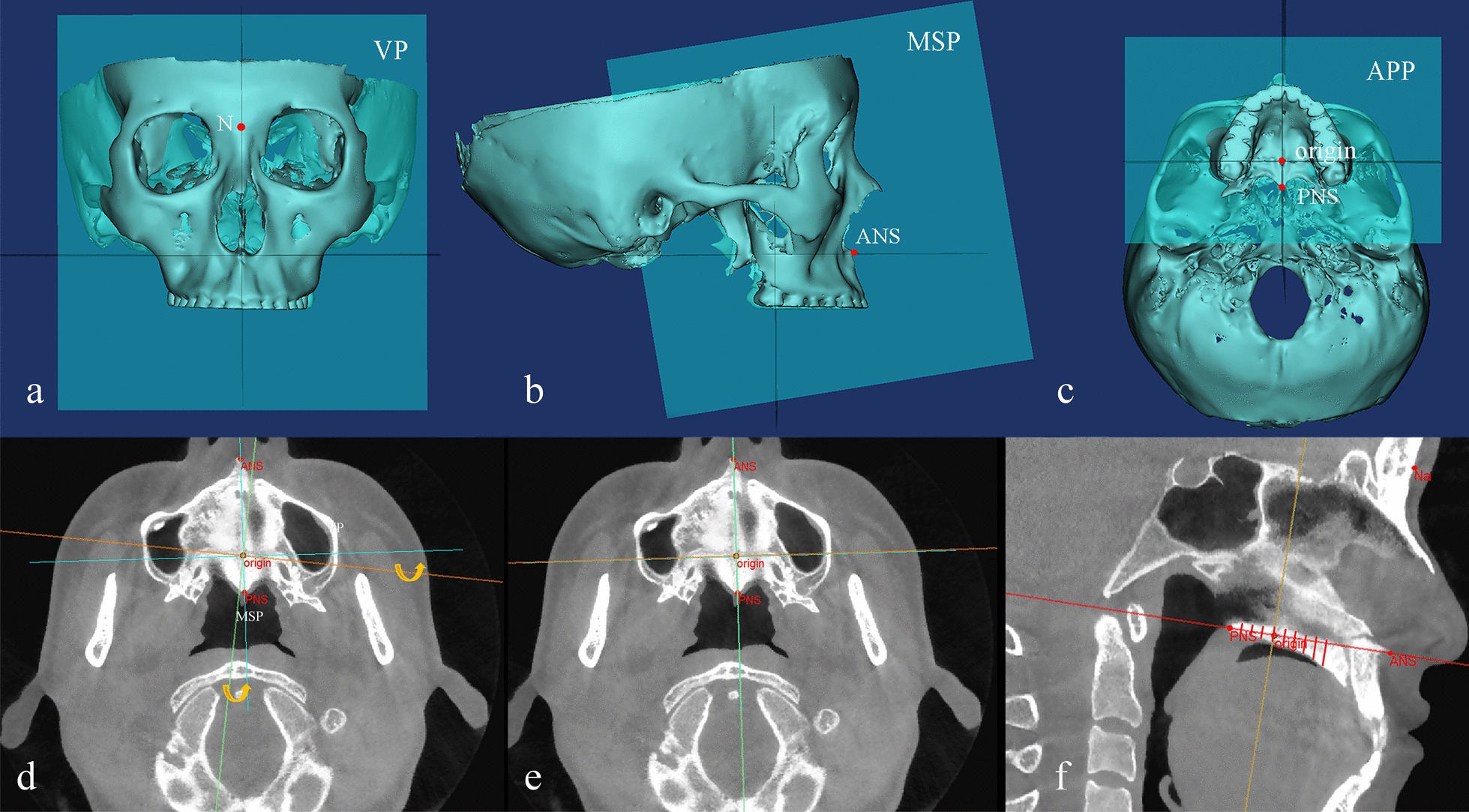
Measurement of the palatal bone thickness. **a**–**c** Three palatal reference planes established in Mimics software; **d**, **e** Images before and after correction in transverse section. The corrections in the sagittal and coronal sections are not shown in the figure; **f** bone thickness at different sites was measured in the sagittal section

The following reference planes were set up in the palate [29] (Fig. 2):

1. *Midsagittal plane (MSP)* the plane passing through the anterior nasal spine (ANS), posterior nasal spine (PNS), and nasion (N).
2. *Axial palatal plane (APP)* the plane passing through the ANS and PNS and perpendicular to the MSP.
3. *Vertical plane (VP)* The point passing through the midsagittal plane and located at the transverse palatine suture is defined as the origin point, and the VP passes through the origin point and runs perpendicular to both the MSP and APP.

**Fig. 3:**
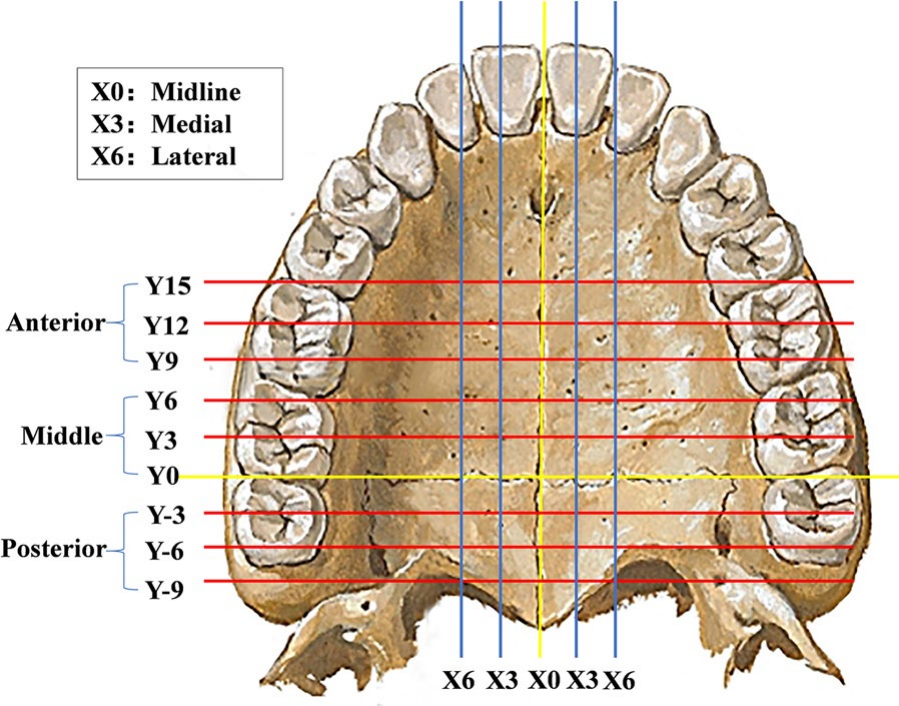
Reference planes for palatal bone thickness measurement

Then, as shown in Fig. 3, coronal planes Y0, Y3, Y6, Y9, Y12, and Y15 parallel to the VP were created 0.0 mm, 3.0 mm, 6.0 mm, 9.0 mm, 12.0 mm, and 15.0 mm in front of the origin. In addition, planes Y-3, Y-6, and Y-9 parallel to the VP were created 3.0 mm, 6.0 mm, and 9.0 mm behind the origin. Sagittal planes X0, X3, and X6 parallel to MSP were created 0.0 mm, 3.0 mm, and 6.0 mm to the left and right sides of the origin. The palatal bone thickness at the junction of each plane was measured. In total, 45 sites were measured.

The measurement items were divided into groups: X0 was defined as the midline area, X3 was defined as the medial area, and X6 was defined as the lateral area. Similarly, there were three regions in the sagittal direction: Y9, Y12, and Y15 were defined as the anterior area; Y0, Y3, and Y6 were defined as the middle area; and Y-3, Y-6, and Y-9 were defined as the posterior area. The “Reslice” function in MIMICS was employed, and the direction of the slices along the reference planes in the views of the coronal section, sagittal section, and transverse section was adjusted. Then, the measurement was performed in the sagittal section (Fig. 2).

### Statistical analysis

All measurements were conducted by a clinician who is familiar with the use of MIMICS software. Two weeks later, 20 patients were selected randomly, and the measurements were repeated by the same clinician. Similarly, another set of 20 patients was randomly selected, and measurements were conducted by another clinician using the same version of MIMICS software on the same computer. SPSS software version 22.0 was used for the statistical analyses. The intraclass correlation coefficients were calculated to evaluate the intra-examiner and inter-examiner reliability. The differences in bone thickness at the same measurement sites between patients with skeletal class III malocclusion and those with skeletal class I malocclusion were determined by independent sample T-tests (for data that were normally distributed and showed homogeneous variance) or Wilcoxon rank-sum tests (for data that did not conform to a normal distribution or exhibited heterogeneity of variance). The bone thickness in the different regions of the palate in the patients with the same malocclusion type was analysed by one-way repeated measures ANOVA. Fisher’s least significant difference (LSD)-t method was used for comparisons in pairs. *P* < 0.05 was defined as the threshold for statistical significance.

## Results

The intraclass correlation coefficients for all measurements with the same examiner and different examiners were greater than 0.9 and 0.85, respectively. This indicated sufficient reliability. In addition, the measurement results revealed no significant differences in the bone thickness measured with respect to the MSP symmetrical plane (*P* > 0.05); therefore, the left and right palatal thickness data were averaged for the subsequent calculations.

### Palatal bone thickness in different regions in the two groups

As shown in Tables 1 and 2, the palatal bone thickness in the patients with skeletal class III malocclusion and those with class I malocclusion showed the same trend. In the sagittal direction, the thickness of the palatal bone was generally lower from front to back in the medial and lateral areas in both groups (*P* < 0.001). In the midline area, the bone is initially thick and then becomes thinner from anterior to posterior (*P* < 0.01). In the coronal direction, the bone thickness of the anterior, middle, and posterior regions of the two groups showed a gradual decrease from the midline area to the medial area to the lateral area, and the bone thickness of the middle area was the greatest. LSD *t* tests showed that the anterior and posterior bone thicknesses were not significantly different in the midline area. Moreover, the differences in bone thicknesses in any two areas in the same plane were statistically significant.

**Table 1:** Palatal bone thickness in patients with skeletal III malocclusion (mm, mean ± standard deviation)

	Midline	Medial	Lateral	F	*P*
Anterior	6.19 ± 1.84^a^	4.41 ± 1.56	3.90 ± 1.74	109.94	*P* < 0.001
Middle	6.75 ± 2.19	3.11 ± 1.12	2.09 ± 1.00	349.36	*P* < 0.001
Posterior	6.29 ± 1.79^a^	2.16 ± 1.02	1.00 ± 0.63	727.03	*P* < 0.001
F	5.31	194.76	250.22		
*P*	*P* = 0.006	*P* < 0.001	*P* < 0.001		

^a^No statistical difference was found (*P* > 0.05)

**Table2:** Palatal bone thickness in patients with skeletal I malocclusion (mm, mean ± standard deviation)

	Midline	Medial	Lateral	F	*P*
Anterior	6.69 ± 1.76^a^	4.98 ± 1.98	4.22 ± 2.09	122.51	*P* < 0.001
Middle	7.73 ± 1.76	3.91 ± 1.42	2.43 ± 1.18	707.98	*P* < 0.001
Posterior	6.63 ± 1.87 ^a^	2.70 ± 1.15	1.25 ± 0.55	660.73	*P* < 0.001
F	28.95	123.96	161.76		
*P*	*P* < 0.001	*P* < 0.001	*P* < 0.001		

^a^No statistical difference was found (*P* > 0.05)

### Palatal bone thickness differences between the two groups

**Fig. 4:**
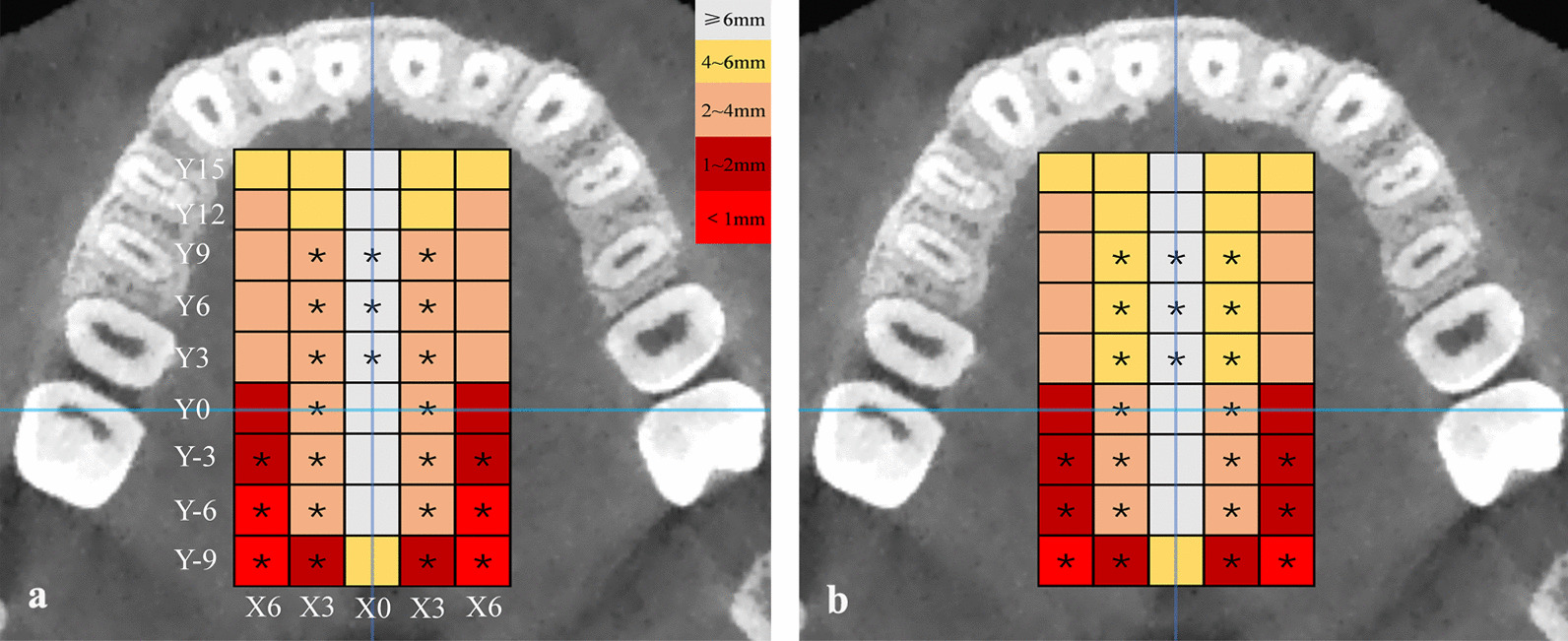
Mean palatal bone thickness maps for a, class III malocclusion and b, class I malocclusion. *Sites have significant differences between the two groups

The differences in palatal bone thickness between patients with skeletal class III malocclusion and those with skeletal class I malocclusion at the same measurement sites are shown in Table 3. In the midline area, the palatal bone of the patients with skeletal class III malocclusion was thinner than that of the patients with skeletal class I malocclusion in three measuring planes (Y3, Y6, and Y9), and the results were statistically insignificant (*P* < 0.05). In the medial area, the palatal bone of the patients with skeletal class III malocclusion in seven planes (from Y-9 to Y9) was comparatively thinner (*P* < 0.05). In the lateral area, the palatal bone in the patients with skeletal class III malocclusion was relatively thicker in the Y-3, Y-6, and Y-9 planes (*P* < 0.05). The palatal bone thickness and regions with significant differences between the two groups were marked with different colours (Fig. 4).

**Table 3:** Results of differences in palatal bone thickness between the two groups (mm, mean ± standard deviation)

	Class III	Class I	Difference (I–III)	*P*
Midline				
Y15	6.55 ± 1.91	7.09 ± 1.87	− 0.53 ± 0.43	0.217
Y12	6.02 ± 1.67	6.41 ± 1.73	− 0.39 ± 0.39	0.316
Y9	6.00 ± 1.91	6.59 ± 1.64	− 0.58 ± 0.40	0.047*
Y6	6.41 ± 2.04	7.38 ± 1.74	− 0.96 ± 0.42	0.026*
Y3	6.83 ± 2.27	7.96 ± 1.79	− 1.13 ± 0.46	0.015*
Y0	7.00 ± 2.28	7.85 ± 1.73	− 0.85 ± 0.45	0.064
Y-3	7.04 ± 1.94	7.35 ± 1.73	− 0.31 ± 0.41	0.452
Y-6	6.44 ± 1.55	6.75 ± 1.89	− 0.31 ± 0.39	0.425
Y-9	5.37 ± 1.46	5.79 ± 1.69	− 0.42 ± 0.36	0.239
Medial				
Y15	5.47 ± 1.63	5.85 ± 2.30	− 0.38 ± 0.45	0.404
Y12	4.16 ± 1.25	4.75 ± 1.73	− 0.59 ± 0.34	0.087
Y9	3.62 ± 1.16	4.34 ± 1.56	− 0.72 ± 0.31	0.022*
Y6	3.40 ± 1.17	4.15 ± 1.45	− 0.76 ± 0.30	0.012*
Y3	3.19 ± 1.08	4.03 ± 1.41	− 0.83 ± 0.28	0.004**
Y0	2.75 ± 1.02	3.56 ± 1.37	− 0.81 ± 0.27	0.004**
Y-3	2.67 ± 1.04	3.40 ± 1.20	− 0.73 ± 0.25	0.005**
Y-6	2.19 ± 0.98	2.71 ± 1.01	− 0.53 ± 0.22	0.021*
Y-9	1.62 ± 0.74	1.99 ± 0.75	− 0.37 ± 0.17	0.029*
Lateral				
Y15	5.19 ± 1.78	5.42 ± 2.38	− 0.23 ± 0.48	0.624
Y12	3.58 ± 1.36	3.99 ± 1.76	− 0.41 ± 0.36	0.251
Y9	2.94 ± 1.23	3.28 ± 1.48	− 0.34 ± 0.30	0.267
Y6	2.41 ± 1.08	2.81 ± 1.34	− 0.40 ± 0.27	0.146
Y3	2.10 ± 0.99	2.45 ± 1.10	− 0.35 ± 0.23	0.141
Y0	1.76 ± 0.82	2.04 ± 0.97	− 0.28 ± 0.20	0.166
Y-3	1.24 ± 0.65	1.62 ± 0.65	− 0.37 ± 0.14	0.012*
Y-6	0.97 ± 0.52	1.11 ± 0.38	− 0.14 ± 0.10	0.040*
Y-9	0.79 ± 0.64	1.03 ± 0.40	− 0.24 ± 0.12	0.048*

*P < 0.05; **P < 0.01

## Discussion

Commonly used clinical maxillary expansion methods include tooth-borne expansion, surgically assisted rapid maxillary expansion (SARME), and miniscrew-assisted maxillary palatal expansion (MARPE) [30–32]. With increasing age, the maxillary suture gradually changes from fibrous bonding to osseous embedding. The traditional tooth-borne expander can therefore no longer be used, and surgical expansion is required [33, 34]. However, SARME induces significant trauma and carries risks such as postoperative infection, pain in the jaw, and neurovascular injury, which also increase the economic burden on the patients [34]. With a success rate of 86.96% in young adults, MARPE has proven to be a feasible treatment option for MTD [35, 36]. MARPE can minimize unnecessary tooth inclination and alveolar bone bending and achieve true skeletal arch expansion [37]. Won Moon and colleagues improved this technique and developed MSEs (Bompole Korea Inc.) based on traditional MARPE [9, 10]. The miniscrews of the MSE are implanted on both sides of the midpalatal suture and placed in the middle and posterior area of the palate, penetrating the double-layer bone cortex for fixation such that the stress of MSE can act on the pear-shaped hole column, zygomatic strut, pterygopalatine suture, and other structures that provide greater resistance to palatine bone expansion [9]. This can lead to a parallel opening of the midpalatal suture in the sagittal direction and effectively expand the entire maxillary complex [10, 29].

In the palatal region included in this study, we found differences in the thickness of palatal bone between patients with skeletal class III malocclusion and those with skeletal class I malocclusion, and the palatal bone of the former was thinner. Piyoros et al. [24] used CBCT to evaluate palatal bone thickness in patients with normal and open vertical skeletal configurations and found that palatal bone thickness was lower at almost all sites in patients with an open bite. These findings suggest that bone thickness is influenced by vertical or sagittal skeletal configurations. The size and shape of the palate are closely related to cranial and maxillofacial morphology. The palatine process is one of the four processes of the maxilla and contributes to the formation of the roof of the mouth and the floor of the nasal cavity [38]. Most patients with skeletal class III malocclusion have maxillary hypoplasia [1], which may be accompanied by palatal hypoplasia. The significant differences in palatal bone thickness between the patients with the two types of malocclusion may be attributable to the influence of palatal growth and development on maxillary growth and development.

Adequate bone volume is needed to ensure the stability of the miniscrews in the MSEs [39]. There were significant differences observed in the area 9.0 mm before the transverse palatine suture in the midline area, 9.0 mm before and after the transverse palatine suture in the medial area, and 9.0 mm after the transverse palatine suture in the lateral area between the two groups (*P* < 0.05). If the implant anchorage is placed in the same area in the two types of patients, the palatal thickness through which the implant penetrates will be different. The thickness is thinner in the patients with class III malocclusion and the minisrews have less contact area with palatal bone, which is an important issue to consider. Ichinohe et al. [43] highlighted that the thickness of the bone cortex in a group with a higher success rate of anchorage screws was significantly greater than that in a group with a lower success rate. The odds ratio for failure of the mini-implant was 6.93 when the cortical bone thickness was less than 1.0 mm, relative to 1.0 mm or more [40]. As shown in Table 3, palatal bone thickness decreased gradually from front to back in the parapalatal and palatal areas, and the mean bone thickness at the sites where the Y-6 and Y-9 planes intersected with X-6 was less than 1 mm. Considering the expansion force of the MSE, the implant anchorage needs to also bear a great force, and the possibility of the loosening of the implant anchorage is greater when the thickness of the palatal bone is insufficient. The implant site of the MSE screws in patients with class III malocclusion may need to be moved forward compared to patients with skeletal I malocclusion.

This study showed that the thickness of palatal bone in the same coronal plane becomes gradually thinner from the middle to the sides. The palatal bone thickness in the midline area is the greatest, and the average values in the front, middle, and back midpalatal sutures are greater than 6.0 mm. Previous studies have also shown that the thickness of the palatal suture in the middle and posterior palatal region in adult patients is larger than that within 6.0 mm on both sides [22, 41, 42]. However, Ichinohe et al. [43] showed that the success rate of implanting screws in the group with a long distance from the midpalatal suture (1.5–2.7 mm) was significantly higher than that in the group with a distance of 0–1.4 mm. This may be associated with the presence of defective calcified areas in the midpalatal suture area. Hence, the bilateral screws of MSE should be implanted symmetrically to the extent possible so that the bilateral implant anchorage is located in the medial area (the distance between the MSE left and right nail holes of approximately 5.0–6.0 mm). Additionally, the palatal bone thickness becomes thinner from the front to back in the midline area. This finding contradicts Poon’s finding that the palatal bone gradually increases in thickness from the front to the back in the midpalatal suture area [41], which may be related to the fact that the other studies considered incisive foramina as the origin, while this study divided the region with the sutura palatina transversa as the centre.

The miniscrew for an MSE is 1.5 mm × 11.0 mm [9]. In theory, the length of the anchorage miniscrew includes the 2.0 mm thickness of the nail hole, 1.0–2.0 mm clearance between arch reamer and palatal mucosa, 1.0–2.0 mm thickness of palatal mucosa, and 5.0–6.0 mm thickness used for double-layer cortical bone binding. This study showed that for patients with skeletal class III malocclusion, the thickness of the palatine bone in the middle and posterior regions of the medial area and the lateral area was less than 4.0 mm, and an 11.0-mm screw would be long enough to penetrate the double cortical bone. However, a thin palatal bone in the middle and posterior regions also increased the risk of penetration of the miniscrew anchorage into the nasal mucosa and even into the inferior turbinate, causing discomfort to patients, and possibly causing local infection and affecting the stability of the implanted screws [44]. Therefore, the size of the MSE screw, especially the length of the two rear implant screws, should be designed more accurately and specifically according to the palatal bone shape and implant direction, and a short implant anchorage should be considered for implantation when necessary [45].

### Limitations

It was an observational comparison study with a small sample size, and patients with an average angle were selected. The representativeness of the sample is limited. Although CBCT has much less ionizing radiation than conventional CT, the patients were still at a risk of exposure to radiation when the CBCT data were being collected.

## Conclusion

The palatal bone was thinner in patients with class III malocclusion than in patients with class I malocclusion and showed significant differences in some areas. These differences in bone thickness should be considered when miniscrews for a MSE are implanted. Palatal bone becomes thinner from front to back and from the middle to both sides within 6 mm on either side of the midpalatal suture. The anterior and middle palatal areas are safer for the implantation of miniscrews. A miniscrew with a length of 11.0 mm increases the risk of penetrating the nasal mucosa and even the inferior turbinate in the posterior area of the palate. Palatal bone thickness should thus be carefully evaluated.

## Data Availability

The datasets used and/or analysed during the current study are available from the corresponding author on reasonable request.

## Abbreviations

CBCT: Cone-beam computed tomography
MTD: Maxillary transverse deficiency
SARME: Surgically assisted rapid maxillary expansion
MARPE: Miniscrew-assisted maxillary palatal expansion
MSE: Maxillary skeletal expander
MSP: Midsagittal plane
APP: Axial palatal plane
VP: Vertical plane
